# Driving Forces Behind the Past and Future Emergence of Personalized Medicine

**DOI:** 10.3390/jpm3010014

**Published:** 2013-01-17

**Authors:** Julius Alexander Steffen, Jan Simon Steffen

**Affiliations:** 1 Imperial College London, Exhibition Road, London SW7 2AZ, UK; 2 Christian-Albrechts-University Kiel, Christian-Albrechts-Platz 4, 24118 Kiel, Germany; E-Mail: jansteffen1@gmx.net

**Keywords:** personalized healthcare, diagnostic testing, genomic medicine, business diversification

## Abstract

Personalized medicine can be seen as a continuously developing approach to tailoring treatments according to the individual characteristics of a patient. In some way, medicine has always been personalized. During the last decade, however, scientific and technological progress have made truly personalized healthcare increasingly become reality. Today’s personalized medicine involves targeted therapies and diagnostic tests. The development of targeted agents represents a major investment opportunity to pharmaceutical companies, which have been facing the need to diversify their business due to an increasingly challenging market place. By investing into the development of personalized therapies, pharmaceutical companies mitigate a major part of the risks posed by factors such as patent expiries or generic competition. Viewing upon personalized medicine from different perspectives points out the multi-causality of its emergence. Research efforts and business diversification have been two main driving forces; they do supplement each other, however, are not jointly exhaustive in explaining the emergence of this approach. Especially in the future, a number of further stakeholders will impact the evolution of personalized medicine.

## 1. Introduction

Presently, personalized medicine is a main issue in all fields of human medicine and subject to scientific, political and economic discussions. As defined by the US Council of Advisors on Science and Technology, “personalized medicine refers to the tailoring of medical treatment to the individual characteristics of a patient” [1]. Recently developed personalized therapies have proven to significantly improve the treatment success in critical fields such as oncology [2,3,4]. Diagnostic tests are used to assess whether or not a targeted treatment will be effective in a certain patient so that the overall patient group is stratified into responders and non-responders [5,6]. This is why Collins describes personalized medicine as “dramatic paradigm shift”, because it bridges the gap from the traditional “one size fits all” approach to a test-and-treat strategy [7]. To explain how this approach has emerged, different perspectives can be taken.

## 2. Personalized Medicine as a Continuously Developing Approach

Instead of viewing personalized medicine as an abrupt innovation, it can be seen as an ongoing process of research efforts made to tailor treatments according to individual patient characteristics. In some way, medicine has always been personalized. For years, physicians have incorporated environmental, behavioral, and genetic aspects that affect risk stratification, drug response or disease management, into their treatment decisions [8]. An example is given by the treatment of hypertension, which is one of the most common diseases worldwide [9]. In many cases, hypertension is treated with beta blockers. However, if the patient also suffers from bronchial asthma, most beta blockers are no treatment option because of their broncho-constrictive side-effects [10]. An ACE-Inhibitor could be a practicable alternative but is contraindicated when the anamneses also contains an angioneurotic edema [11]. Consequently, physicians have to take the individual characteristics of hypertension patients into consideration and tailor their treatments accordingly. 

What has been changing during the last decade, however, is the technology and tools which are available to physicians. The ability to sequence the human genome at rapidly decreasing costs has led to a better understanding of various diseases and has facilitated the development of novel diagnostic technologies and pharmacotherapeutic strategies [12,13,14]. Today’s personalized medicine therefore adds a new factor to the set of characteristics according to which a treatment can be tailored: the individual genetic and molecular profile. Knowing that a certain genetic alteration drives the proliferation of a disease, a targeted treatment which is particularly effective in the respective patient group can be developed. Also, this knowledge can be used to identify the responders to targeted treatments in advance, save costs and time, and spear patients the side-effects of treatment failures [5,12,13,15]. The identification of treatment responders, in turn, requires a diagnostic test, which is why today’s personalized medicine can be defined as tandems of therapeutic and diagnostic product [13,15]. An early but very impressive example of this approach is the clinical management of the chronic myeloid leukemia (CML) (Box 1).

The currently most common approach to diagnostic testing is called companion diagnostics, which implies that each targeted treatment is accompanied by a corresponding diagnostic test [16]. As more disease-driving mutations are identified, more diagnostic tests can potentially be conducted for one patient. This leads to a high consumption of material and time and a multiplication of costs, which might ultimately be a major impediment on the further development and implementation of personalized treatments [17]. In the near future, however, diagnostic testing can be expected to occur in a parallel way [13,18]. Technologies such as multiplex assays and whole-genome sequencing make the simultaneous assessment of multiple genetic mutations in a single test possible and thus prevent a multiplication of costs [17].

**Box 1. The personalized treatment of chronic myeloid leukemia (CML).**

CML is one of the most common malignancies in the group of myeloproliferative neoplasias. Initially described by Rudolf Virchow in 1845 [19], CML has an incidence of about 2 cases per 100,000 per year [20].

Over a long time, chemotherapies using hydroxyurea or busulfan were carried out to reduce the disease symptoms of CML whereas the introduction of stem-cell-transplantation and the treatment with interferon-α were the only options to slow down progression or achieve remission of CML [20]. The success of these therapeutic strategies, however, was increasingly limited.

CML is based on a reciprocal translocation of t(9; 22) (q34; q11), resulting in a fusion of the BCR- and the ABL-gene [21]. The fusion gene encodes a tyrosine kinase, which can be held responsible for deregulating the cell-cycle and affecting DNA-repair [20].

Based on the molecular-genetic understanding of pathomechanisms concerning the BCR-ABL protein, Imatinib (Glivec®) was brought to the pharmaceutical market in 2001. Imatinib is a selective tyrosine kinase inhibitor (TKI), which targets the tyrosine kinase BCR-ABL1 [22]. This very effective pharmacon enables physicians to successfully treat a remarkably large group of CML patients [22].

The example of Imatinib is a step of outstanding importance towards personalized medicine. Its impact, however, depends on a particular form of the translocation 9/22. If the specific genetic alteration is absent the drug is non-effective, but if the translocation is present it is highly effective and represents a major improvement as compared to conventional therapies [22].

By examining whether or not a CML patient harbors the translocation, the treatment success can be predicted prior to the actual treatment. Therefore, a Fluorescence in situ hybridization is conducted as a diagnostic test, stratifying the overall patient group into responders and non-responders.

Recently, the development of second-generation TKIs has opened up advanced perspectives of a personalized treatment, taking account of patient-characteristics like comorbidity or mutated forms of the BCR-ABL-gene, which can be easily detected due to innovative laboratory techniques [23].

The goal of further research is to realize the entire scope of personalized medicine. Besides the identification of treatment responders, truly personalized medicine will encompass four additional aspects. These can be arranged in chronological order, starting with risk assessment. Based on genomic knowledge, genetic tests can assess the susceptibility of an individual to a certain disease. Subsequently, prevention can take place, involving changes in behavior, lifestyle and treatment to prevent a disease. If risk assessment and prevention have not taken place successfully, molecular tests help detect diseases at an early stage, before symptoms become observable. After the diagnosis of the disease development and the according treatment, the final step of personalized medicine is management. This involves active monitoring of disease progression [13].

## 3. Personalized Medicine as a Business Diversification Strategy

Personalized medicine can also be described as a corporate growth strategy properly addressing the challenges posed by past developments in the drug market. Patent expiries, generic competition, little R&D success and political restrictions have rendered the market place more challenging, so that pharmaceutical companies have been forced to re-evaluate their strategy and find new ways towards corporate growth [24]. 

The traditional growth strategy of Research and Development (R&D) oriented pharmaceutical companies has been based on the “blockbuster model”. The basic idea of this strategy is that large revenues are generated by a small number of products. A blockbuster describes a drug that reaches annual revenues of above $US 1 billion [25]. Pfizer Inc. has been a typical example of that strategy: In 2009, Pfizer spent $US 7.7 billion for R&D. IBM, in comparison, spent $US 5.8 billion [26]. Still, Pfizer obtained only one US patent for every 10 US patents IBM obtains. This does not mean that Pfizer’s R&D projects are less effective. It can rather be seen as a characteristic of the pharmaceutical industry that the total amount of R&D expenses is allocated to a relatively low number of patents. The blockbuster strategy therefore aims at launching products which generate sufficient profits to compensate for the entire R&D expenses [25]. 

Until 2015, however, six out of the ten largest pharmaceutical companies will suffer patent expiries accounting for more than 50% of total sales in 2009 [24]. Additionally, nearly 50% of pharmaceutical executives have been expecting negative returns on their R&D investments [24]. This is reinforced by political restrictions involving utilization reviews and cost controls which are aimed at restricting market access in response to exploding health care costs [24]. Against this background, the majority of pharmaceutical executives have been regarding business diversification as a potential strategic alternative [24]. 

Diversification can generally be defined as the introduction of new products in new markets. To the majority of pharmaceutical companies, personalized medicine represents a diversification strategy. First, because they did not offer any personalized therapies before and second because the markets which are relevant to personalized therapies were often not represented in their product portfolio before. By developing a personalized therapy in Non-Small-Cell Lung Cancer, for instance, a traditional pharmaceutical company introduces a new therapy to a new market and thus diversifies its business. 

The reason why personalized medicine has become increasingly appealing is that it is less affected by the current market challenges. Patent expiries of conventional drugs, for instance, lead to a rapid increase in generic competition, which causes significant revenue cuts. Personalized medicine, on the other hand, comprises targeted therapies with a specific mode of action which can hardly be copied by competing generic manufacturers. Additionally, personalized medicine is a field with only few existing therapies so that the chance of successful R&D projects is clearly higher than in conventional fields. And lastly, the therapeutic improvements realized by personalized medicine will be a major asset when it comes to reimbursement negotiations. 

**Figure 1:**
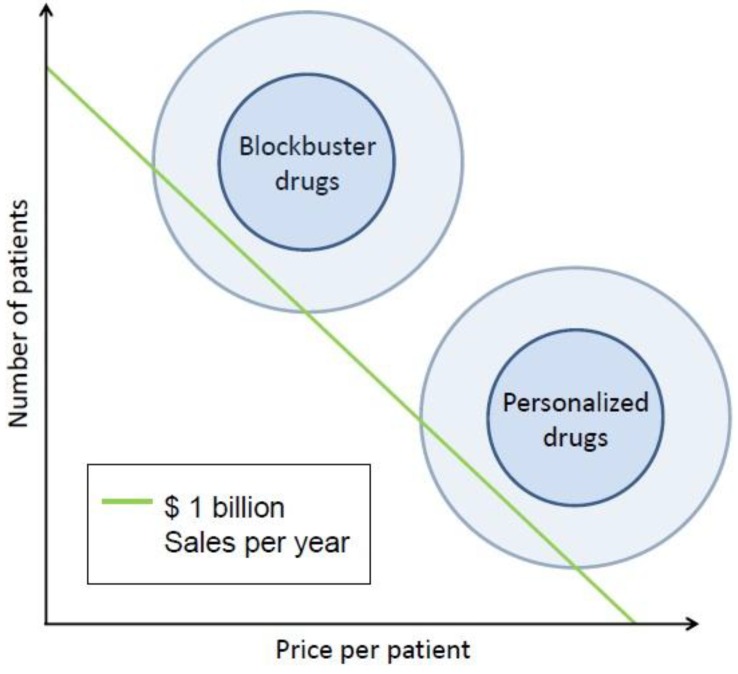
Volume-based revenues of traditional blockbuster drugs *vs*. margin-based revenues of personalized medicine drugs.

Diversifying into the market of personalized medicine is linked to a particular revenue model: whereas blockbuster drugs are launched in high-volume markets, personalized therapies have a more specifically defined target group marked by a lower number of patients. Revenues generated in the field of personalized medicine are therefore driven by margins instead of volumes. Both revenue models can theoretically generate the same amount of revenues (Figure 1). In light of the current blockbuster crisis, personalized medicine is therefore a highly attractive alternative to traditional growth strategies.

In this perspective, the emergence of personalized medicine correlates with the corporate strategies of pharmaceutical companies. By making major investments into the research and development of personalized medicine, pharmaceutical companies majorly increase their availability and thus drive their emergence. 

## 4. An Integrated Perspective

Looking upon personalized medicine from different perspectives makes clear that the emergence of this approach is not mono-causal. In fact, on-going research efforts and business diversification supplement each other and add up to a bigger picture of how personalized medicine has emerged. 

Contrasting different perspectives suggests that there has been a logical sequence in which personalized medicine has evolved. Traditional treatment tailoring on the basis of general patient characteristics did not require the existence of targeted drugs developed by pharmaceutical companies, yet. However, by discovering concrete genetic abnormities which cause the proliferation of a disease, scientists have been able to define molecular structures which can be targeted by respective drugs. Through this, they have created an investment opportunity for pharmaceutical companies. By investing into the development of targeted therapies, pharmaceutical companies are nowadays playing a key role as they increase the availability of personalized therapies and make them accessible for a larger patient group. 

As personalized medicine has increasingly emerged, the required efforts have been growing above and beyond the capabilities of research bodies and pharmaceutical companies. Today’s status quo of personalized medicine requires the engagement of diagnostic companies as a further stakeholder. To successfully provide a tandem of therapeutic and diagnostic product, a respective test must be developed in a simultaneous manner, suggesting a coordination of diagnostic and pharmaceutical R&D activities. This further implies that the regulatory processes for diagnostic and therapeutic products must coincide [27]. Although the US Food and Drug Admission has released guidance for companion diagnostics in 2011, the Personalized Medicine Coalition criticizes that “major logical difficulties” in the co-development process have not yet been resolved [28].

Besides diagnostic companies and regulatory bodies, payers can be added as a further stakeholder having a major impact on the further evolution of personalized medicine. After all, a successful implementation of today’s and future personalized treatments requires an appropriate reimbursement structure. As companion diagnostics represent a novel field, the lack of adequate reimbursement schemes has frequently been criticized [5,29]. The current reimbursement infrastructure in most European countries fails to properly assess the value provided by combinations of drug and diagnostics [29].

Finally, pathologists will have to be added as a further significant stakeholder group. As the future success of personalized medicine will heavily depend on the availability and implementation of sequencing technologies, the traditional role of pathologists will have to be redefined [30]. While this is already true of today’s clinical practice, it will increasingly apply to the future. With technologies such as whole-genome sequencing becoming available, large amounts of new data will be generated. As part of their critical task of translating these technologies into clinical practice, pathologists will have to be capable of judging when data displaying genetic abnormities give reason for the diagnosis of a disease [31].

The integrated perspective has significant implications on how personalized medicine will further be developed in the future. It suggests that partnerships between purely scientific research bodies, regulatory agencies, politics, as well as pharmaceutical and diagnostic companies will be the key success factor. No single stakeholder will be able to set all the necessary requirements to further drive the emergence and evolution of personalized therapies [32]. The value of this approach can already be observed in today’s clinical practice, for example, during the current implementation of parallel testing technologies. To fully capture the benefits of these powerful technologies, it will be critical to create a proper testing infrastructure. As exemplified by the Center for Integrated Oncology in Cologne, this will require the creation of regional testing networks [33,34]. This concrete and very successful example highlights the importance of political stakeholders but also suggests that the efforts of clinics and hospitals will be of major importance.

## 5. Conclusions

Integrating different perspectives provides a more exhaustive view, suggesting that scientific research and corporate diversification do not exclude but actually supplement each other. Additionally, the integrated perspective also points out that both factors do not suffice in fully explaining the emergence of personalized medicine. This is especially true of the present and the future environment, which is heavily impacted by further stakeholders such as regulatory agencies, political bodies and payers. 

In the end, personalized medicine is a multi-facetted approach requiring the coordination of multiple stakeholders. This has been true of its past emergence and will particularly apply to its future development and implementation. 
